# Patterns of Twitter Behavior Among Networks of Cannabis Dispensaries in California

**DOI:** 10.2196/jmir.7137

**Published:** 2017-07-04

**Authors:** Nicholas C Peiper, Peter M Baumgartner, Robert F Chew, Yuli P Hsieh, Gayle S Bieler, Georgiy V Bobashev, Christopher Siege, Gary A Zarkin

**Affiliations:** ^1^ RTI International Behavioral Health and Criminal Justice Research Division Research Triangle Park, NC United States; ^2^ RTI International Center for Data Science Research Triangle Park, NC United States; ^3^ RTI International Survey Research Division Research Triangle Park, NC United States; ^4^ RTI International Research Computing Division Research Triangle Park, NC United States

**Keywords:** cannabis, marijuana, social networking, social media, Internet

## Abstract

**Background:**

Twitter represents a social media platform through which medical cannabis dispensaries can rapidly promote and advertise a multitude of retail products. Yet, to date, no studies have systematically evaluated Twitter behavior among dispensaries and how these behaviors influence the formation of social networks.

**Objectives:**

This study sought to characterize common cyberbehaviors and shared follower networks among dispensaries operating in two large cannabis markets in California.

**Methods:**

From a targeted sample of 119 dispensaries in the San Francisco Bay Area and Greater Los Angeles, we collected metadata from the dispensary accounts using the Twitter API. For each city, we characterized the network structure of dispensaries based upon shared followers, then empirically derived communities with the Louvain modularity algorithm. Principal components factor analysis was employed to reduce 12 Twitter measures into a more parsimonious set of cyberbehavioral dimensions. Finally, quadratic discriminant analysis was implemented to verify the ability of the extracted dimensions to classify dispensaries into their derived communities.

**Results:**

The modularity algorithm yielded three communities in each city with distinct network structures. The principal components factor analysis reduced the 12 cyberbehaviors into five dimensions that encompassed account age, posting frequency, referencing, hyperlinks, and user engagement among the dispensary accounts. In the quadratic discriminant analysis, the dimensions correctly classified 75% (46/61) of the communities in the San Francisco Bay Area and 71% (41/58) in Greater Los Angeles.

**Conclusions:**

The most centralized and strongly connected dispensaries in both cities had newer accounts, higher daily activity, more frequent user engagement, and increased usage of embedded media, keywords, and hyperlinks. Measures derived from both network structure and cyberbehavioral dimensions can serve as key contextual indicators for the online surveillance of cannabis dispensaries and consumer markets over time.

## Introduction

Dramatic population-based shifts in cannabis use have occurred over the past 15 years in the United States [1]. As of July 2017, 29 states and Washington DC have enacted laws that permit medical cannabis use. Much research has helped to understand individuals who use cannabis for medical purposes [2], ranging from their consumption patterns and motivations for use to service satisfaction and clinical preferences [3-6]. Similar efforts have explored how state laws differentially impact operation and enforcement of cannabis businesses, health centers, and cultivation practices [7,8]. Nevertheless, significant public debate remains about the medicinal value of cannabis given the large body of clinical and population-based studies showing increased risk of many adverse outcomes [8], especially with regard to the effects of high potency strains and concentrated products like edibles [9,10].

Notably, these debates coincide with the growing availability of medical and recreational cannabis products at dispensaries across the United States. In California, the world’s largest legal market for cannabis, medical cannabis patients report that they vary their purchasing behaviors based upon product pricing and availability at dispensaries as well as the specific conditions for which they received a physician recommendation [11]. Patients also report that experiences and interactions with dispensary staff like budtenders greatly influence their purchasing behaviors, including their willingness to try new products [12]. Because dispensaries serve as the purveyors of cannabis products and strongly influence population-based consumption [11,12], many advertise their products and services through a wide variety of platforms, including social network platforms like Twitter.

While it is estimated that 1 in 2000 tweets pertain to cannabis [13], there are currently no studies that specifically focus on how dispensaries use Twitter to engage with medical cannabis patients and their larger follower base. This gap is particularly salient as content analyses of influential Twitter users show that cannabis-related tweets tend to elicit positive sentiments towards cannabis, including heavy and frequent use behaviors [13]. A recent study found that WeedTweets (@stillblazingtho), a Twitter account with over 1 million followers, posts an average of 10 tweets per day and that these tweets tend to normalize regular cannabis use, especially among youth and certain minority populations [14]. In addition, other studies have detected higher frequencies of tweets related to cannabis concentrates (eg, edibles, dabs, and oils) in states that allow medical and recreational consumption [15,16], which may be partially attributable to increasingly permissive and accepting attitudes toward cannabis.

Considering the well-documented impacts of social networks on consumer preferences and behaviors [17-19], an explicit focus on the exchange of cannabis-related information may provide valuable insights into how networks of cannabis consumers form around dispensaries on Twitter. For instance, some dispensaries regularly use Twitter to share their menus, inform their followers about new products, offer coupons and promotions, promote retail services, post industry trends and events, and mention findings from scientific studies. Other dispensaries, however, may engage in these practices less frequently or have more sporadic Twitter usage, which could influence their ability to form strong and sustained networks of followers.

More importantly, systematic investigation of dispensaries on Twitter can provide insights into how dispensaries behave on the Internet and how these behaviors influence the formation of shared follower communities. This comparative study therefore examines a set of 12 Twitter cyberbehaviors among two samples of cannabis dispensaries from the San Francisco Bay Area (SFBA) and Greater Los Angeles (GLA). For each metropolitan area, we visualize overall network structure and community formation based upon shared followers, then reduce the cyberbehaviors into a more parsimonious set of dimensions with principal components factor analysis. Finally, we utilize quadratic discriminant analysis to investigate whether the extracted dimensions of cyberbehavior significantly differentiate between dispensary communities in California.

## Methods

### Study Sample

We adapted aspects of targeted sampling methods to select cannabis dispensaries in SFBA and GLA. Traditionally, these methods have been used in social science and public health studies to access “hidden” populations (eg, medical cannabis patients or people who inject drugs) outside of community or medical settings [20]. Targeted sampling integrates components of street ethnography, theoretical sampling, stratified survey sampling, quota sampling, and respondent-driven sampling [21-24]. As it improves upon convenience samples through a purposive and rigorous process, a growing body of studies has used targeted sampling to recruit representative samples that are comparable to those achieved through random sampling techniques [25-28].

In the context of cannabis dispensaries in California, we included licensed, registered, and commercially zoned dispensaries from the Medical Dispensary Program in San Francisco, the Cannabis Regulatory Commission in Oakland, the Medical Cannabis Commission in Berkeley, and the Medical Marijuana ID Program in Los Angeles. We then cross-referenced the initial database with Leafly, WeedMaps, and THCFinder, three popular cannabis sites that allow users to geolocate dispensaries throughout the United States, including California. These sites include streamlined platforms with comprehensive information about social network profiles, which allowed us to expand our initial database and create a larger catchment of dispensary accounts on Twitter. With this final sample of dispensary accounts, we collected the account IDs of followers and the last 3200 tweets available as of February 16, 2016. Finally, a set of 12 cyberbehaviors were derived with metadata from the accounts and fell into three broad categories: account age, posting frequency, and tweet composition (Table 1).

**Table 1:** Definitions for Twitter cyberbehaviors.

Cyberbehaviors		Definition
**Account age**		
	Overall age	Number of days a Twitter account has existed
	Total days tweeting	Number of days at least one tweet was sent from an account
**Posting frequency**		
	Tweets collected	Total number of tweets collected from an account timeline
	Percentage of days tweeting	Percentage of days since an account was created that there has been a tweet
	Max. tweets per day	Maximum number of times an account has posted a tweet in a single day
	Average tweets per day	Mean number of times an account tweets per day^a^
	Median absolute deviation	Median absolute deviation (MAD) of tweets per day
**Tweet composition**		
	Hashtag (#)	Percentage of tweets collected that contained a hashtag
	Mention (@)	Percentage of tweets collected that mentioned another user directly
	Retweet (RT)	Percentage of tweets collected that were retweets
	Media	Percentage of tweets collected that contained embedded media^b^
	Hyperlink (http://)	Percentage of tweets collected that contained a hyperlink

^a^Excludes days on which an account did not tweet.

^b^Images, videos, and documents.

### Network Structure and Community Detection

**Figure 1:**
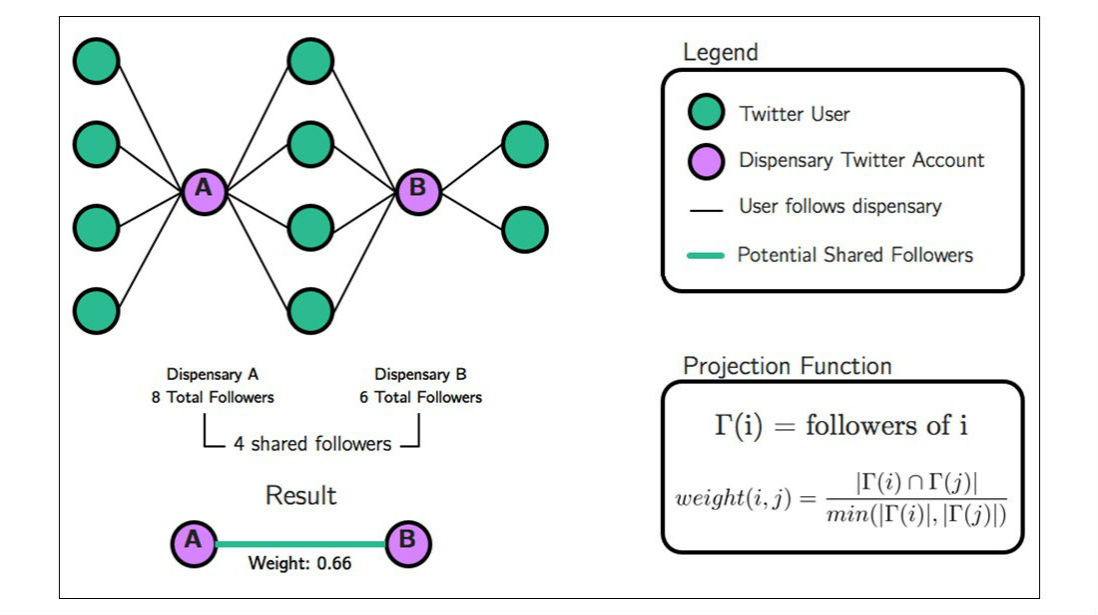
Hypothetical shared follower network.

With the account information for each dispensary and their followers, we created a projection from the dispensary networks with edge weights representing shared followership [29]. Because the sampled dispensary accounts had a highly right-skewed distribution of followers, we normalized follower counts between two dispensaries by calculating the ratio of shared followers to potential shared followers, where *potential shared followers* was the minimum of the follower counts of the two dispensaries. With similarities to the Jaccard index, this measurement computed a projection function that determined the shared potential followers between dispensaries [30,31]. As depicted in Figure 1, two hypothetical dispensaries share four followers out of a total of six potential shared followers, yielding a projection function of 66%.

The Louvain Method [32] was then utilized to detect dispensary communities in SFBA and GLA. This unsupervised algorithm finds communities of large networks and provides a hierarchical structure for the network through an iterative, two-stage process that maximizes modularity. The method first began by selecting a random dispensary (ie, node) and assigning that dispensary to a community of one of its neighboring dispensaries, until all existing dispensaries in the network were assigned to a community. In the second phase, each dispensary represented a community from phase one, while edges between dispensaries represented the sum of the weights of the previous connections between dispensaries in those two communities. These two phases of optimizing modularity and constructing a meta-network in each city were repeated until a network with the maximum value of modularity was found.

After creating the network data and calculating communities, we visualized each city’s network with a force-directed graph drawing algorithm. This network visualization algorithm places nodes with more shared follower potential closer to each other and repulses nodes with limited or no potential. For the purposes of this study, dispensaries from the same community were visualized using colored nodes.

### Cyberbehavioral Dimensions and Community Classification

For the 12 cyberbehaviors, descriptive statistics were computed to characterize each city’s set of communities. Wilcoxon rank-sum and Kruskal-Wallis tests were also performed to explore any statistically significant differences between cities and communities for the 12 cyberbehaviors. We then conducted principal components factor analysis (PCA) with a 12x12 correlation matrix of the cyberbehaviors to extract empirically meaningful dimensions. PCA provided a method with which to address multicollinearity among the cyberbehaviors and arrive at a more parsimonious set of dimensions that account for the data variability. Lastly, we performed quadratic discriminant analysis (QDA) to determine the classification accuracy of the extracted cyberbehaviors [33]. For the purpose of this study, QDA produced classification tables for each city, which allowed for distinguishing between modularity classes with the extracted cyberbehavioral dimensions from the PCA.

## Results

### Descriptive Statistics

Overall, a total of 119 dispensary accounts were examined, with 61 in SFBA and 58 in GLA. The mean values for each cyberbehavior are shown for the two cities in Table 2. Each account in SFBA and GLA was approximately three years old on average. The cyberbehaviors for posting frequency and tweet frequency were highly comparable between the two cities. Although dispensary accounts in SFBA spent a higher number of days tweeting and sent more tweets than accounts in GLA, no significant differences were found.

### Network Structure and Community Formation

**Figure 2:**
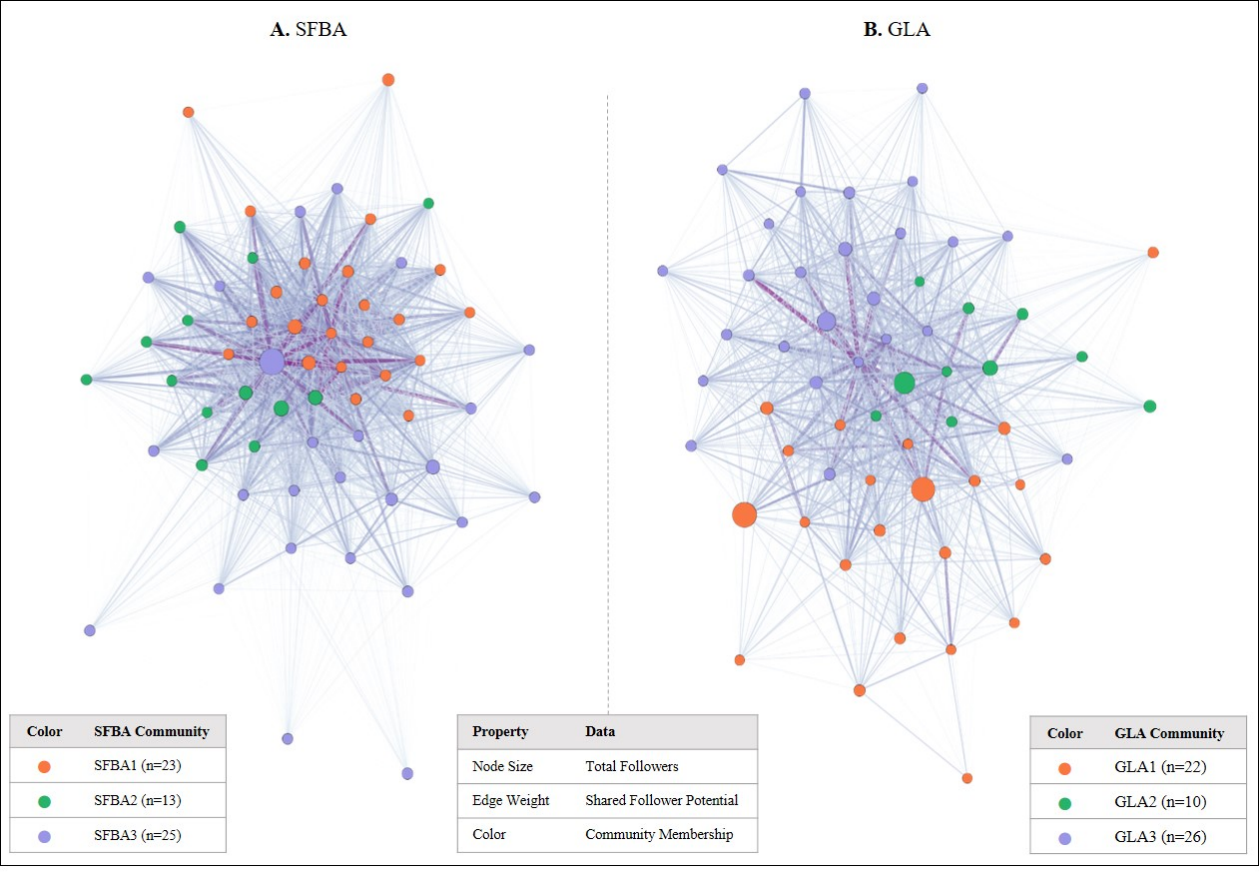
Shared follower networks in the San Francisco Bay Area and Greater Los Angeles.

Figure 2 visualizes the two shared follower networks of dispensaries in SFBA and GLA. The size of the nodes corresponds to the total number of followers, the thickness of the edges indicates the shared follower potential, and the color of the nodes refers to the community classifications from the Louvain method. Overall, the distribution of shared followers between each pair of accounts differed between SFBA and GLA. The range of shared followers was .2% to 71% in SFBA compared with 3% to 46% in GLA.

Among the SFBA networks, 21% (n=13, marked in green) of dispensaries were in a weakly connected community with all members having a modest number of Twitter followers. Another 38% (n=23, marked in orange) were in a fairly centralized community of dispensaries with strong interconnections between smaller accounts. The largest community accounted for 41% of the sample (n=25, marked in purple) and had strong interconnections through the most popularly followed dispensary. In GLA, a community accounting for 38% (n=22, marked in orange) of the network had the two dispensaries with the most followers, although its members were weakly connected. A small and weakly connected network accounted for 17% of the network (n=10, marked in green), with only two that had a relatively large group of followers. Despite only a modest number of followers on Twitter, the remaining 45% of the network (n=26, marked in purple) formed the largest and most strongly connected community, indicating a substantial portion of shared followers between any given pair of members.

**Table 2:** Descriptive statistics for Twitter cyberbehaviors.

Cyberbehaviors	SFBA^a^ (n=61)	GLA^b^ (n=58)	*P* value^c^
	Mean	Mean	
Account Age, Days (Years)	1107.8 (3.0)	1006.2 (2.8)	.49
Total Days Tweeting	285.5	202.6	.14
Tweets Collected	965.4	590.3	.21
Max. Tweets Per Day	15.1	16.4	.87
Average Tweets Per Day	3.0	2.9	.72
MAD^d^ Tweets Per Day	0.8	0.8	.92
Percentage of Days Tweeting	25.9	24.1	.34
Percentage of Tweets with Media	20.4	21.0	.98
Percentage of Tweets with #^e^	40.4	40.4	.92
Percentage of Tweets with @^f^	26.1	27.6	.54
Percentage of Tweets with RT^g^	10.2	10.5	.63
Percentage of Tweets with Hyperlink	55.9	51.8	.47

^a^SFBA: San Francisco Bay Area.

^b^GLA: Greater Los Angeles.

^c^The *P* values were calculated with the Wilcoxon rank-sum tests to accommodate for the nonparametric nature of the cyberbehaviors.

^d^MAD: median absolute deviation.

^e^#=hashtag.

^f^@=user mention.

^g^RT: Retweet.

**Figure 3:**
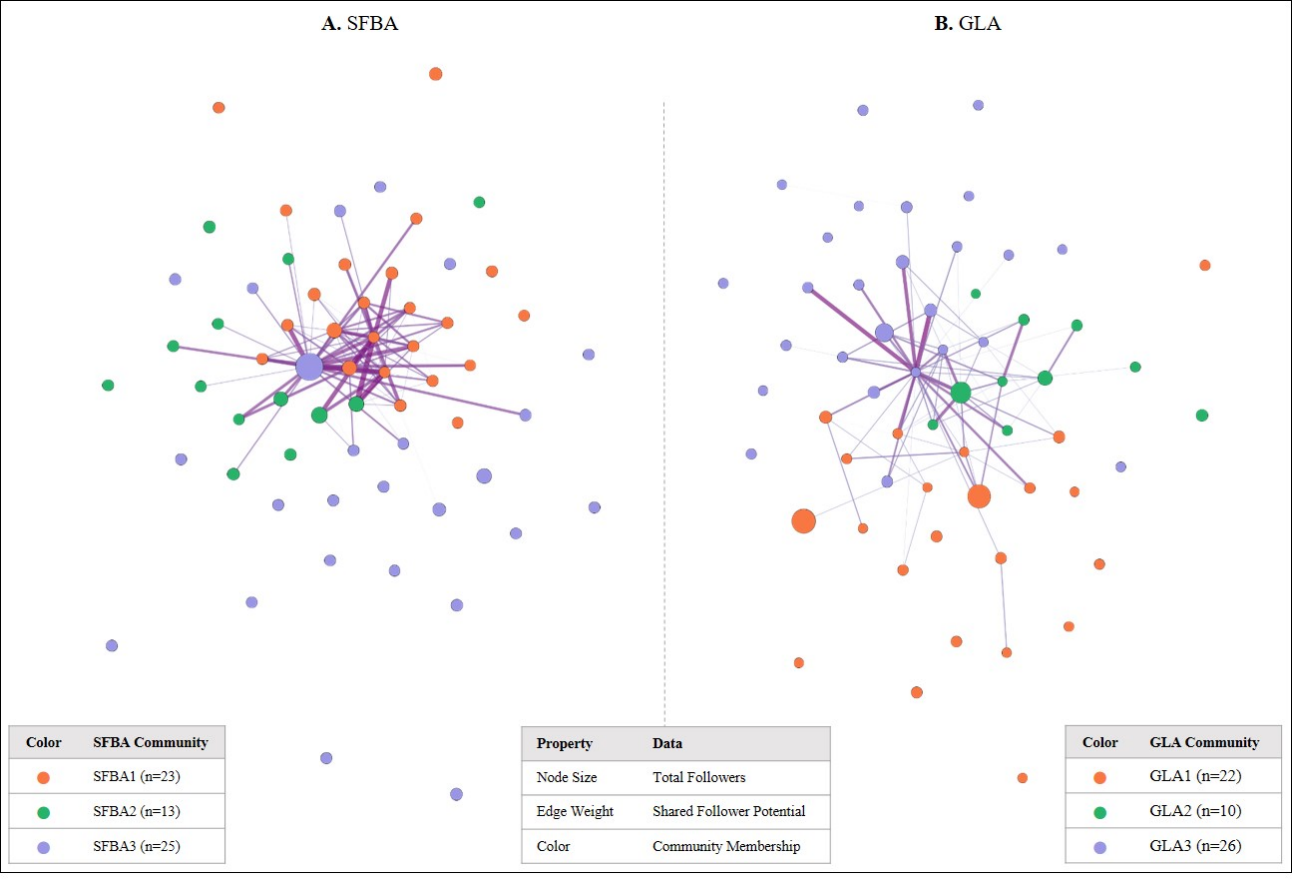
Shared follower network subgraphs in the San Francisco Bay Area and Greater Los Angeles.

In the subgraphs (Figure 3), we recalibrated the tie-strength and considered an edge to be present when the proportion of shared followers of a given pair of dispensaries was above the 95th percentile of shared follower potential between any given pair of dispensaries in each of the two cities. We tested various thresholds of shared follower potential: the median, the third quartile (75th percentile), 90th percentile and 95th percentile. As highly consistent network graphs were found, the 95th percentile was used as the final threshold to produce the subgraphs with the strongest tie-strength in the social networks with the best visual clarity.

As Figure 3 illustrates, one dispensary is particularly popular in SFBA, where it not only attracts substantially more followers than its counterparts, but also has stronger connections to the followers. In contrast, large dispensaries in GLA are not as strongly connected and centralized as those in SFBA, given that they do not share many followers (Figure 3). Although four of them seem to be very popular with a large group of followers, they occupy different network positions and attract different groups of Twitter users through a smaller but highly centralized and interconnected dispensary. Additionally, there was a cluster of well-connected dispensaries with a group of small, but mostly shared followers.

### Cyberbehavioral Dimensions

The mean values for the cyberbehaviors are summarized for the extracted communities in Multimedia Appendix 1. In SFBA, the weakly connected orange community had lower rates of maximum tweets per day, average tweets per day, hashtags, and user mentions, despite having the highest number and percentage of days where a tweet was sent. In comparison, the moderately connected green and strongly connected purple communities had higher frequencies of tweets as well as more user engagement (eg, mention and retweet), hashtag usage, embedded media, and hyperlinks. Significant differences between SFBA communities were found for account age, total days tweeting, average tweets per day, and percentage of tweets with media. For the GLA dispensaries, the highly connected purple community had the highest percent of days tweeting, embedded media, hashtag usage, and hyperlinks. The weakly connected green and orange communities tended to have higher account ages, total tweet days, and total tweets. The only significant differences between GLA communities were found for account age.

The principal components factor analysis yielded five relevant factors (eigenvalues>1.0) that describe the Twitter behaviors of dispensaries in SFBA (Table 3). The first factor for SFBA was classified as *activity* (eigenvalue=4.3) and included three behaviors indicating the daily message frequency of users: maximum tweets per day, average tweets per day, and median absolute deviation of tweets per day. The second factor, *age* (eigenvalue=3.2), included two items: account age and percentage of tweets with media like images and videos, suggesting a specific Twitter usage pattern among SFBA dispensaries with older accounts that were less likely to include media in their tweets.

We categorized the third factor for SFBA dispensaries as *longevity* (eigenvalue=1.4) with both total days tweeting and percentage of days tweeting loading on to this dimension, followed by *engagement* (eigenvalue=1.2) with both percentage of tweets that were retweets (ie, tweets being forwarded or shared with others by users who read the original tweet) and mentions (ie, including another user account in the tweet) being loaded on this factor. The engagement dimension captures how users interact with each other on Twitter. Lastly, *referencing* (eigenvalue=1.0) included two items that link the tweet to additional information sources: percentage of tweets with hashtags (#) and hyperlinks (http://).

**Table 3:** Results from principal components factor analysis of the 12 cyberbehaviors in the San Francisco Bay Area.

Cyberbehaviors			Activity		Age		Longevity		Engagement		Referencing
Eigenvalues^a,b^			4.3		2.2		1.4		1.2		1.0
	Account Age	0.02		**0.60**		−0.02		0.01		−0.18	
	Total Days Tweeting	−0.06		0.29		**0.54**		−0.05		−0.13	
	Percentage of Days Tweeting	−0.04		−0.20		**0.74**		0.06		0.12	
	Tweets Collected	0.28		0.19		0.32		−0.11		−0.03	
	Max. Tweets Per Day	**0.46**		0.03		0.07		0.10		−0.01	
	Average Tweets Per Day	**0.64**		−0.03		−0.15		−0.06		0.02	
	MAD^c^ Tweets Per Day	**0.53**		−0.05		0.05		0.06		0.02	
	Percentage of Tweets with Media	0.05		− **0.64**		0.14		−0.07		−0.29	
	Percentage of Tweets with #^d^	−0.03		−0.21		−0.06		0.16		**0.55**	
	Percentage of Tweets with @^e^	0.01		−0.01		0.02		**0.68**		−0.10	
	Percentage of Tweets with RT^f^	−0.01		0.07		0.02		**0.68**		0.05	
	Percentage of Tweets with Hyperlinks	0.03		0.14		0.10		-0.11		**0.73**	

^a^The presence of dimensionality was supported when eigenvalues were 1.0 or greater. Values for each cyberbehavior are expressed as varimax-rotated factor loadings.

^b^Bold factor loadings denote values greater than or equal to .40.

^c^MAD: median absolute deviation.

^d^#=hashtag.

^e^@=user mention.

^f^RT: Retweet.

We found highly similar cyberbehavioral dimensions among the GLA dispensaries (Table 4). The factor that explained the most variance was *activity* (eigenvalue=3.0), including average tweets per day and median absolute deviation of tweets per day with the largest loadings. We classified the second factor as *longevity* (eigenvalue=2.4), given that account age, total days tweeting, and total number of tweets collected significantly loaded on to this dimension. The third factor found from the GLA data was *engagement* (eigenvalue=1.8), with the same two behavioral items being loaded on to this dimension. A similar *referencing* dimension was found (eigenvalue=1.3) in GLA, although hashtags were accompanied with a significant loading for percent tweets with multimedia content when compared with hyperlinks in SFBA. For GLA, a significant loading for *hyperlinks* formed its own dimension (eigenvalue=1.3).

**Table 4:** Results from principal components factor analysis of the 12 cyberbehaviors in Greater Los Angeles.

Cyberbehaviors	Activity	Longevity	Engagement	Referencing	Hyperlinks
Eigenvalues^a,b^	3.0	2.4	1.8	1.3	1.2
Account Age	0.14	**0.48**	0.05	0.26	0.29
Total Days Tweeting	0.10	**0.66**	0.01	0.08	0.08
Percentage of Days Tweeting	0.16	0.20	0.25	0.39	0.29
Tweets Collected	0.22	**0.54**	0.00	0.02	0.01
Max. Tweets Per Day	0.33	0.05	0.14	0.26	0.32
Average Tweets Per Day	**0.64**	0.03	0.02	0.06	0.12
MAD^c^ Tweets Per Day	**0.59**	0.03	0.05	0.08	0.09
Percentage of Tweets with Media	0.11	0.01	0.01	**0.62**	0.21
Percentage of Tweets with #^d^	0.08	0.00	0.19	**0.54**	0.09
Percentage of Tweets with @^e^	0.01	0.00	**0.66**	0.13	0.01
Percentage of Tweets with RT^f^	0.00	0.03	**0.66**	0.07	0.01
Percentage of Tweets with Hyperlinks	0.11	0.00	0.01	0.04	**0.80**

^a^The presence of dimensionality was supported when eigenvalues were 1.0 or greater. Values for each cyberbehavior are expressed as varimax-rotated factor loadings.

^b^Bold factor loadings denote values greater than or equal to .40.

^c^MAD: median absolute deviation.

^d^#=hashtag.

^e^@=user mention.

^f^RT: Retweet.

### Classification Accuracy of Cyberbehavioral Dimensions

Table 5 illustrates how the communities classified by the cyberbehavioral dimensions (ie, columns) corresponded to the true communities identified through the Louvain Method (ie, rows). As depicted by the bolded diagonals, the dimensions correctly classified 75% (46/61) of the dispensary communities in SFBA. The orange community had the best classification precision, followed by the green and purple communities.

In GLA, the dimensions correctly classified 71% (41/58) of the dispensary communities, with high classification precision among the orange and purple communities (Table 6). Only 20% of the green community was correctly classified, most likely due to limited sample size. Additional loading statistics for the dimensions in the QDA may be found for each city in Multimedia Appendix 2 and interpreted like the factor loadings from the PCA.

**Table5:** Classification table for the communities of dispensaries in the San Francisco Bay Area.

San Francisco Bay Area (N=61)	Classified community		
True community	Orange	Green	Purple
Orange (n=23)	**20** **(87%)^a^**	0 (0%)	3 (13%)
Green (n=13)	2 (15%)	**9** **(69%)**	2 (15%)
Purple (n=25)	3 (12%)	5 (20%)	**17** **(68%)**

^a^Bold diagonals illustrate correctly classified communities.

**Table6:** Classification tables from the quadratic discriminant analysis of dispensaries in Greater Los Angeles.

Greater Los Angeles (N=58)	Classified community		
True community	Orange	Green	Purple
Orange (n=22)	**18** **(82%)^a^**	0 (0%)	4 (18%)
Green (n=10)	3 (30%)	**2** **(20%)**	5 (50%)
Purple (n=26)	4 (15%)	1 (4%)	**21** **(81%)**

^a^Bold diagonals illustrate correctly classified communities.

## Discussion

### Principal Findings

As a popular social network platform that enables rapid information exchange about controversial social phenomena, Twitter represents an unregulated domain where cannabis dispensaries can form communities through regular communication and engagement with large audiences. In this study, the networks in SFBA and GLA both included sets of highly influential dispensaries with large groups of shared followers. However, the network structure of SFBA was more strongly connected and centralized than that of GLA, which had four large dispensaries that occupied relatively separate network spaces. The most strongly connected dispensaries in both cities had newer accounts, higher daily activity, more frequent user engagement, and increased usage of embedded media, keywords, and hyperlinks. As such, both network structure and cyberbehaviors significantly distinguished between the communities in each city, which provides evidence for contextual indicators that can be utilized for the surveillance of information exchange among dispensaries on Twitter.

### Cyberbehaviors and Distinguishable Communities­

Among the large and interconnected dispensary communities, the cyberbehaviors indicated regular tweets to shared followers that may include patient, consumer, and cannabis industry populations with strong mutual interests. The younger age of these highly active dispensaries may also demonstrate the emergence of new marketing strategies that streamline product promotions, share information, and develop brand loyalty within a larger sharing economy on Twitter [34]. In addition, these communities exhibited comparatively higher user engagement and referencing, two dimensions that may reciprocate collective consumption of cannabis through Twitter-mediated interactions and cooperative cyberbehaviors that rapidly disseminate cannabis-related information [35]. Together, the structural and dimensional characteristics of these communities indicate that influential dispensaries may use Twitter to boost social traffic to their websites and grow their social networks [18,36-41].

In contrast, the dispensaries on the network periphery had lower shared follower potential and exhibited more generic cyberbehaviors (eg, text-only tweets) that do not provide followers with engaging content or links to additional resources. As populations with greater socioeconomic status are significantly more likely to send and receive hyperlinks [42-45], these dispensaries may lack the resources to engage in cyberbehaviors that place them in more densely connected network spaces characterized by regular communications and strategic engagements with larger populations of shared followers. The *referencing* and *hyperlink* dimensions found in this study may therefore serve as key contextual measures of social capital among cannabis markets in California. Indeed, several large dispensaries were able to occupy their own network spaces outside of the center cores in both cities through increased *referencing* and *hyperlinks*, which may help attract shared followers with regional preferences, motivations, and norms related to cannabis consumption [46]. As several California studies have found that dispensaries are more likely to cluster in communities with higher levels of cannabis demand, consumption, and morbidity [47-50], follow-up analyses that incorporate geospatial data will be better suited to determine how network position corresponds to the geographic distribution of dispensary communities in California and other states [51].

With regard to community formation among dispensaries, we conceptualized shared followers as a form of affinity that signals mutual interest and affiliation with dispensaries they choose to jointly follow. By incorporating this feature into a social network to understand interconnections between dispensaries, shared followers represent a potential resource that may flow between dispensaries and help form communities in response to unique patterns of cyberbehavior among dispensaries [52-54]. In the larger context of public health surveillance, the social networks constructed in SFBA and GLA may serve as the foundation for more rigorous studies to evaluate how new social policies and regulations disrupt or facilitate community formation and cyberbehavior. Moreover, the rapidly growing presence of dispensaries on the Internet suggests that the cyberbehaviors identified in this study may be useful measures to capture the frequency and types of communication that occur on Twitter [55,56]. Coordinated efforts to engage with researchers, policymakers, and stakeholders will be necessary to better understand the utility of these measures and develop scalable strategies to monitor large-scale industry practices on Twitter and other social media platforms [57,58].

### Limitations

Although this study utilized shared follower potential to understand network structure and community formation among dispensaries, we acknowledge the multiple ways in which social networks may be represented. Instead of shared follower networks, a strict flow network using shared or liked tweets among dispensaries may demonstrate different dynamics of social interaction and information propagation [59]. Indeed, exploratory analyses revealed very low levels of message diffusion among dispensaries in SFBA and GLA, which suggests that shared followers typically do not exchange or directly engage with dispensary tweets. Considering the referencing dimension, the content from dispensary tweets may also be constructed as a semantic network that not only illustrates conceptual connections between phrases, keywords, and hashtags, but also classifies how cannabis products are priced and promoted [60,61]. While such analyses were beyond the scope of this paper, rigorous content analyses will provide the framework with which to create a classification system that can be systematically trained to identify direct-to-consumer advertising of cannabis products and other specific types of tweets, such as health claims, industry events, scientific studies, and sentiment towards state and federal policies [61-63].

Similarly, the ability of the cyberbehavioral dimensions to distinguish between communities suggests that metadata can provide additional insights into dispensary and consumer behaviors on Twitter. Larger studies that leverage these dimensions with metadata like dispensary type (eg, nonprofit, delivery, and health services), provisions for state and local laws, and geospatial characteristics may improve the detection of dispensary communities [64]. For example, computational methods like stochastic block modeling can improve the accuracy of community detection with metadata without *a priori* assumptions about their correlations [65]. In other words, these methods can learn (eg, unsupervised and semi-supervised) whether important correlations exist and subsequently use or ignore metadata depending on whether they provide useful information to network structure and community formation [65]. Finally, integrative techniques like exploratory graph analysis, latent network modeling, and residual network modeling represent new and exciting approaches that can help derive more parsimonious cyberbehavioral dimensions when compared with PCA and other latent variable modeling approaches [66,67].

### Conclusions

The findings from this study indicate that network structure and multiple dimensions of dispensary behavior on Twitter shape two of California’s largest cannabis markets. As California successfully passed Proposition 64 on the November 2016 ballot, the legalization of recreational cannabis use for adults aged 21 years and older further stresses the need to determine the policy implications of online cannabis marketing and monitor community activity through contextual measures of cyberbehavior that may influence population-based consumption. In addition, the emergence of online marketplaces and mobile apps demonstrates how the digitization of dispensaries has started to shift consumers away from storefronts to high-tech collaborative consumption platforms that personalize product choices and automate purchases. With Twitter as a key part of this digital paradigm shift, the scalable methodology used in this study will serve as the basis for more rigorous designs that longitudinally track community formation and patterns of cyberbehavior among dispensaries.

## Appendix

Multimedia Appendix 1 Descriptive statistics for cyberbehaviors among communities of dispensaries.

Multimedia Appendix 2 Canonical loadings for Twitter cyberbehaviors among communities of dispensaries.

## Abbreviations

GLA: Greater Los Angeles
PCA: Principal components factor analysis
QDA: Quadratic discriminant analysis
SFBA: San Francisco Bay Area
