# Supplementary material for: Patterns of Twitter Behavior Among Networks of Cannabis Dispensaries in California
Source: J Med Internet Res. 2017 Jul 4;19(7):e236. doi: 10.2196/jmir.7137 (PMC5516098; doi:10.2196/jmir.7137)
Supplement: Multimedia Appendix 1 [file jmir_v19i7e236_app1.pdf]

Cyberbehaviors	Orange (n=23)	Green (n=13)	Purple (n=25)	
SFBA (N=61)[a]	Mean	Mean	Mean	*P* value[c]
Account Age, Days (Years)	1224.4 (3.4)	1690.0 (4.6)	697.9 (1.9)	<.001
Total Days Tweeting	390.6	364.9	147.6	.01
Tweets Collected	1061.7	1482.5	608.0	.08
Max. Tweets Per Day	11.6	21.6	14.8	.07
Average Tweets Per Day	2.3	3.6	3.2	.04
MAD[d] Tweets Per Day	1.7	2.2	2.4	.57
Percentage of Days Tweeting	29.9	18.8	25.8	.24
Percentage of Tweets with Media	17.6	9.1	28.7	.01
Percentage of Tweets with #[e]	35.7	40.8	44.5	.49
Percentage of Tweets with @[f]	21.4	29.1	28.9	.54
Percentage of Tweets with RT[g]	5.7	14.7	12.0	.15
Percentage of Tweets with Hyperlink	52.0	50.1	62.4	.41

	Orange (n=22)	Green (n=10)	Purple (n=26)	
GLA (N=58)[b]	Mean	Mean	Mean	*P* value[c]
Account Age, Days (Years)	1618.5 (4.4)	961.3 (2.6)	505.4 (1.4)	0.001
Total Days Tweeting	267.1	239.9	133.8	0.16
Tweets Collected	736.2	909.9	344.0	0.17
Max. Tweets Per Day	16.0	23.1	14.3	0.95
Average Tweets Per Day	2.6	3.5	2.9	0.97
MAD[d] Tweets Per Day	0.6	1.1	0.8	0.89
Percentage of Days Tweeting	15.9	20.3	32.6	0.08
Percentage of Tweets with Media	14.7	19.7	26.9	0.11
Percentage of Tweets with #[e]	35.0	39.4	45.4	0.39
Percentage of Tweets with @[f]	28.6	24.0	28.2	0.72
Percentage of Tweets with RT[g]	12.2	12.0	8.6	0.49
Percentage of Tweets with Hyperlink	43.7	49.1	59.7	0.19

[a]Abb: San Francisco Bay Area.

[b]Abb: Greater Los Angeles.

[a]The *P* values were calculated with Kruskal-Wallis tests to accommodate for the nonparametric nature of the cyberbehaviors.

[d]Abb: Median Absolute Deviation.

[e] #=hashtag.

[f] @=user mention.

[g] Abb: Retweet.
