# Supplementary material for: Patterns of Twitter Behavior Among Networks of Cannabis Dispensaries in California
Source: J Med Internet Res. 2017 Jul 4;19(7):e236. doi: 10.2196/jmir.7137 (PMC5516098; doi:10.2196/jmir.7137)
Supplement: Multimedia Appendix 2 [file jmir_v19i7e236_app2.pdf]

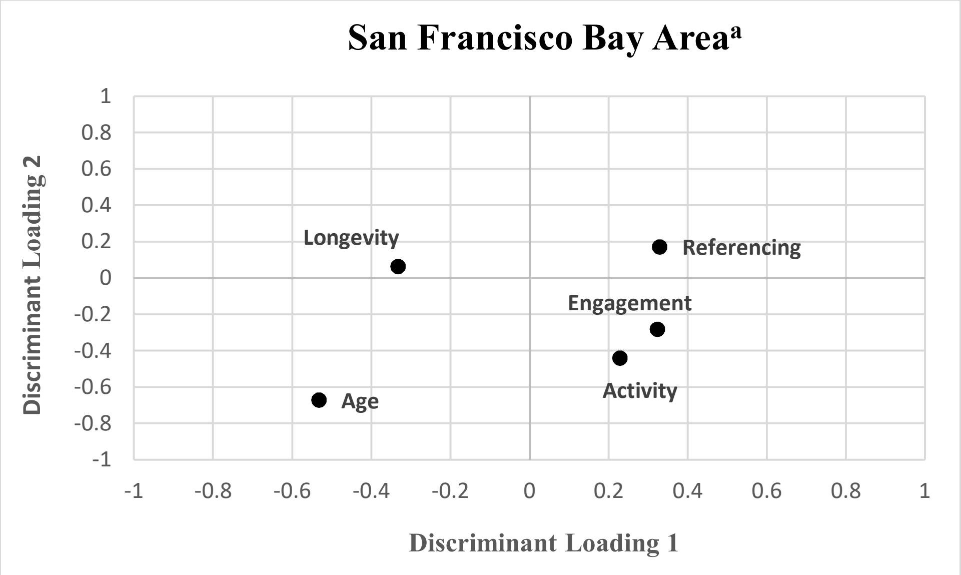

[a]The two discriminant functions were both highly significant (*P's* < 0.001). The first function had a canonical correlation of 0.55 and accounted for 57% of the variance; and the second function had a correlation of 0.49 and accounted for the remaining 43% of the variance. The figure above shows the canonical loadings for the five dimensions. *Age* had the highest absolute loadings for both discriminant functions, while *activity* had the highest absolute loading for the second function. *Engagement* and *referencing* had modest absolute loadings for both functions.

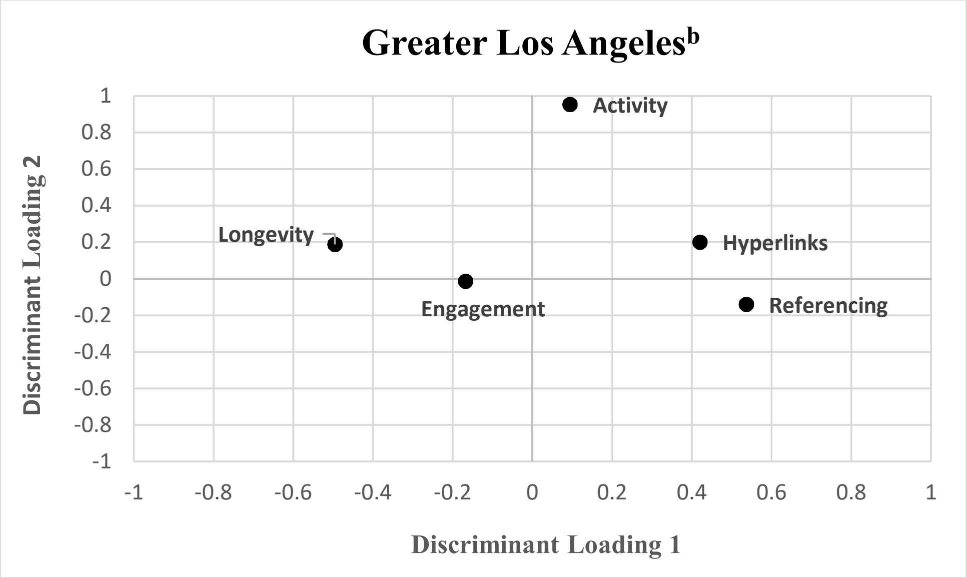

[b]The first discriminant function was highly significant ($F_{10,102}$=3.51, $P < .001$) and accounted for 95% of the variance. The canonical correlation was 0.65 compared to 0.20 for the second function, which did not reach significance ($F_{4,52}$=0.55, $P = .70$). *Longevity*, *referencing* and *hyperlinks* significantly distinguished between communities of dispensaries in Greater Los Angeles.
